# Trends and Future Perspectives of Polysaccharide-Based Bigels from Seeds, Vegetable Oils, and Waxes: A Bibliometric Review

**DOI:** 10.3390/gels11060413

**Published:** 2025-05-30

**Authors:** Monserrat Sanpedro-Díaz, Alitzel Belem García-Hernández, Ana Luisa Gómez-Gómez, Julia Salgado-Cruz, Oswaldo Arturo Ramos-Monroy, Rubén Oliver-Espinoza, Griselda Argelia Rivera-Vargas, Ma de la Paz Salgado-Cruz

**Affiliations:** 1 Escuela Nacional de Ciencias Biológicas, Instituto Politécnico Nacional, Ciudad de México 07738, Mexico; msanpedrod1700@alumno.ipn.mx (M.S.-D.); agomezg1900@alumno.ipn.mx (A.L.G.-G.); oramosm@ipn.mx (O.A.R.-M.); griverav@ipn.mx (G.A.R.-V.); 2 SECIHTI—Centro de Investigación en Química Aplicada, Parque de Investigación e Innovación Tecnológica, Apodaca 66628, Nuevo León, Mexico; ali_ialee@outlook.com; 3 Ingeniería Aeronaútica, Universidad Politécnica de Apodaca, Apodaca 66600, Nuevo León, Mexico; 4 Centro de Investigación Especializado en el Desarrollo de Tecnologías de la Información y Comunicación, Ciudad de México 14050, Mexico; julia.salgado@teit.cfe.mx; 5 Centro de Investigaciones Económicas, Administrativas y Sociales, Instituto Politécnico Nacional, Ciudad de México 11360, Mexico; roliver@ipn.mx

**Keywords:** oleogel, hydrogel, plant polysaccharides, seed, application, food science

## Abstract

Gels are semi-solid colloidal systems characterized by three-dimensional networks capable of retaining up to 99% of liquid while exhibiting both solid-like and liquid-like properties. A novel biphasic system, the bigel, consists of hydrogel and oleogel, enabling the encapsulation of hydrophilic and lipophilic compounds. Their structure and functionality are influenced by the distribution of gel phases (e.g., oleogel-in-hydrogel or hydrogel-in-oleogel). This study aims to review current trends in polysaccharide-based bigels derived from seeds, vegetable oils and waxes, highlighting their biocompatibility, sustainability and potential food applications. A bibliometric analysis of 157 documents using VOSviewer identified four key thematic clusters: structured materials, delivery systems, pharmaceutical applications, and physicochemical characterization. Principal component analysis revealed strong correlations between terms, while also highlighting emerging areas such as 3D printing. This analysis demonstrated that seed-derived polysaccharides, including chia seed mucilage and guar gum, improve bigel structure and rheological properties, offering sustainable plant-based alternatives. Additionally, innovations such as extrusion-based 3D printing, functional food design, controlled drug release, bioactive compound delivery, and fat replacement are helping to support the further development of these systems. Finally, bibliometric tools remain instrumental in identifying research gaps and guiding future directions in this field.

## 1. Introduction

Growing concerns regarding the adverse health effects of saturated fatty acids (SFAs) and trans fatty acids (TFAs), particularly in solid and semi-solid forms, have driven efforts to develop healthier lipid alternatives. These fats, traditionally used in bakery, meat, and confectionery products, play crucial structural and sensory roles [1,2]; however, excessive consumption has been strongly associated with increased risks of cardiovascular disease, hypertension, and obesity [3]. Despite these concerns, replacing SFAs and TFAs remains challenging due to their essential functional properties in food systems.

Recent advances have focused on designing substitutes that retain desirable product qualities while improving nutritional profiles. This requires not only functional efficacy but also sustainability and scalability. In this context, structured gels, such as hydrogels, oleogels, and bigels, have emerged as promising fat replacers, effectively mimicking the physicochemical and functional properties of conventional solid fats.

Gels are colloidal semisolid systems characterized by a three-dimensional polymeric network capable of retaining large amounts of liquid, often up to 99% of their total mass [4,5,6,7]. Depending on the nature of the matrix, gels exhibit a range of physicochemical properties, making them exceptional materials that combine rigidity and elasticity. This dual behaviour endows gels with both solid-like and liquid-like characteristics to gels [8].

**Figure 1:**
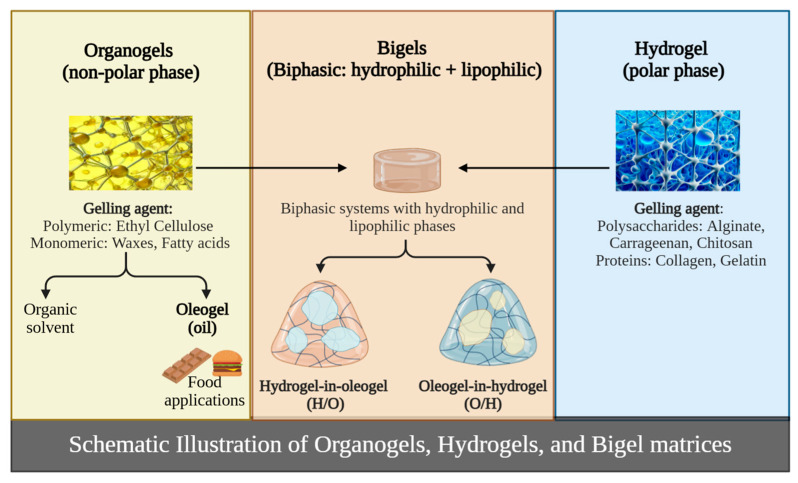
Representation of hydrogel/oleogel matrices combined into a bigel system (Created in BioRender: (https://app.biorender.com; accessed on 15 March 2024) Scientific Image and Illustration Software).

Owing to these unique properties, gels have emerged as versatile materials with diverse applications across industries, including food science, pharmaceuticals, cosmetics, and biomaterials [4,9,10]. Structurally, gels are composed of two main phases: a liquid phase either polar, as in hydrogels, or nonpolar, as in organogels or oleogels, which acts as a gelling agent, and a continuous solid phase. The gelling agent stabilizes the three-dimensional network and imparts the characteristic semisolid consistency to the system (Figure 1) [11].

When organogels are composed entirely of edible components, they are referred to as oleogels. In recent decades, oleogels have attracted increasing attention in the food industry as potential solid fat replacers, offering promising alternatives for structuring oils in trans-fat-free formulations. However, despite their advantages, oleogels alone may not always achieve the optimal balance of mechanical properties, stability, and functionality required for specific food applications [12,13,14,15].

To overcome these limitations and enhance their functional performance, hydrogels and oleogels can be combined to form biphasic semisolid systems known as bigels or hybrid gels [13,16]. Bigels are advanced delivery systems that integrate the structural features of both hydrophilic and hydrophobic gels, resulting in biphasic networks capable of encapsulating and releasing both water-soluble and fat-soluble active compounds [17].

Bigels are classified based on the spatial arrangement of their gel phases (Figure 1). In oleogel-in-hydrogel (O/H) systems, the nonpolar phase is dispersed within a continuous polar matrix. In contrast, in hydrogel-in-oleogel (H/O) systems, the polar phase is dispersed within a nonpolar continuous matrix [4,6]. Although bigels share similarities with emulsions, they differ fundamentally in their semisolid consistency, which imparts unique physicochemical properties and enhances stability [4,6,18].

Polysaccharide-based bigels derived from seeds, vegetable oils and waxes have recently gained attention in food science due to their biocompatibility, sustainability and potential for healthier product development. This article aims to review the current knowledge on hydrogels, oleogels and bigels with a focus on emerging trends and perspectives. Bibliometric analysis is a key tool for understanding research, identifying gaps and guiding innovation. These insights support strategic decision-making for stakeholders.

## 2. Results and Discussion

### 2.1. Bibliometric Analysis of Gels, Bigels, Oleogels and Hydrogels

#### 2.1.1. Keyword Co-Occurrence Analysis with VOSviewer

**Figure 2:**
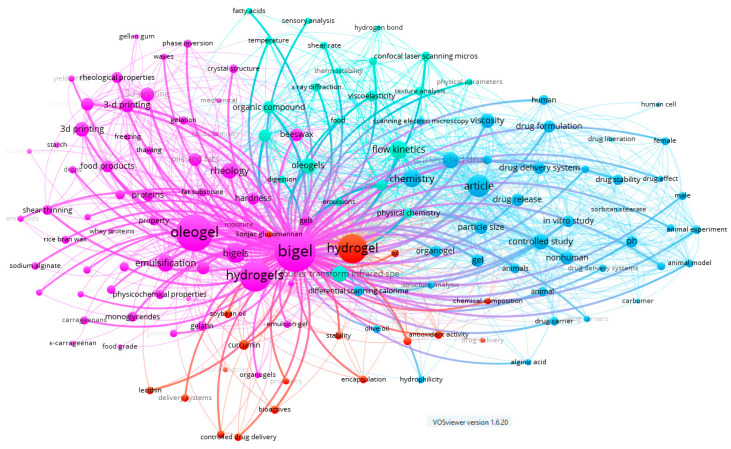
Network map of keywords co-occurrence. Each node represents a specific keyword, while the colours indicate distinct thematic clusters identified through co-occurrence analysis. The edges between nodes reflect the frequency and strength of co-occurrence relationships among the keywords.

A comprehensive keyword analysis was carried out by evaluating the 1540 keywords extracted from the 157 documents included in the study. This analysis aimed to identify trends within the proposed research topic. The co-occurrence map reveals four thematic clusters, as shown in Figure 2.

The first cluster prominently features the terms bigel and oleogel, represented as the largest nodes on the map. This indicates that these terms are among the most studied and interconnected concepts within the network. Other significant terms, including hydrogels, 3D printing, rheology, emulsification, and beeswax, highlight a growing interest in the development of structured materials and their applications in food and biomaterials.

The second cluster is centred around the term “hydrogel”, with research trends focusing on encapsulation [19], antioxidant capacity [20,21,22,23], stability, bioactives [5,7,11], and controlled drug delivery [24,25,26,27,28,29,30].

The third cluster, represented by the term ‘article’, highlights themes such as drug release, drug delivery systems, controlled trials, drug stability, drug formulation, etc. These findings underscore the relevance of gelled systems in pharmaceutical and biomedical applications [24,25,26,27,31].

Finally, the fourth cluster includes terms like flow kinetics, shear rate, texture analysis, viscoelasticity, and temperature. These terms are closely related to the physicochemical and stability characterization of these systems.

Notably, keywords such as ‘3D printing’, ‘organic compounds’, also exhibit high relevance, reinforcing the multidisciplinary nature of this research field and its applications across food science as fat replacers [12,14], 3D printing technologies [11,17,32,33,34] and pharmacology [24,27,28,29,35].

#### 2.1.2. Principal Component Analysis (PCA) of Keywords

**Figure 3:**
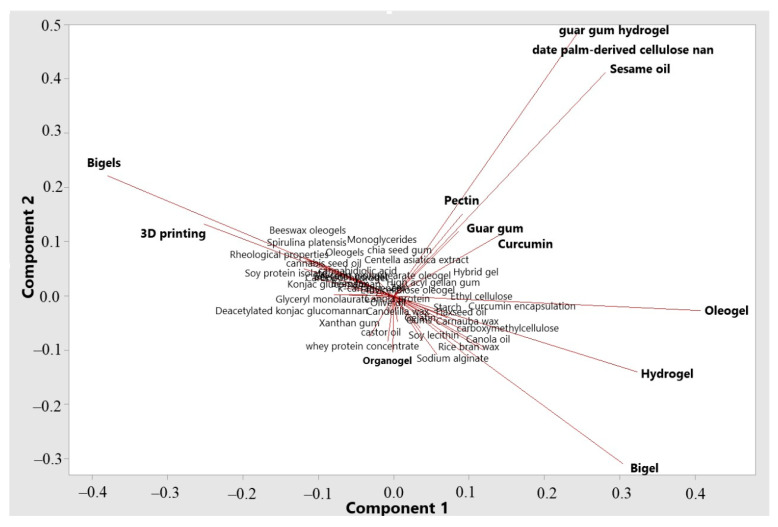
PCA Biplot: Principal Component Analysis of Keywords.

The biplot generated through Principal Component Analysis (PCA, Figure 3) illustrates the impact of specific keywords in bigel research while reducing the dimensionality of variables. This graph provides crucial insights into term correlations: vectors (lines) with small angles indicate strong correlation, those pointing in the same direction reflect positive correlation, while vectors in opposite directions indicate a negative correlation. Right angles indicate no correlation between terms.

The biplot shows that the terms ‘bigel’, ‘hydrogel’, and ‘oleogel’ have long vectors pointing in the same direction, suggesting strong correlations and their status as interconnected concepts within the research field. In contrast, ‘3D printing’ and ‘bigels’ point in different directions, indicating they represent distinct approaches yet remain interrelated. This indicates their significance and representativeness in the analyzed studies.

A cluster of terms, including ‘guar gum hydrogel’ and ‘sesame oil’ are also observed, which are far from the central group, highlighting their contributions to data variability in different directions. The association of bigels with 3D printing signals an emerging trend exploring advanced technologies. Meanwhile, terms such as ‘guar gum hydrogel’ and ‘curcumin’ align more closely with functional and pharmaceutical applications, while ‘oleogel’ and ‘hydrogel’ are often oriented towards structural properties and food applications.

#### 2.1.3. Polysaccharides from Seeds: Emerging Sustainable Alternatives

Bigels combine the advantageous properties of hydrogels and oleogels; however, the materials used in their formulation largely dictate their functionality and applications. Gelling agents, also known as gelators, can be classified based on various criteria, including molecular weight (low vs. high), origin (natural vs. synthetic), and solvent type (hydrogel vs. organogel) [4,36]. These classifications underscore the wide-ranging properties and applications of gelling agents.

Low-Molecular-Weight Gelators (LMWGs) are small molecules, typically with molecular weights below 1 kDa, that form gels through non-covalent interactions such as hydrogen bonding, van der Waals forces, π–π stacking, and hydrophobic interactions [37]. These mechanisms drive the self-assembly of fibrillar structures, creating supramolecular networks capable of entrapping solvents. LMWGs are notable for their efficiency, requiring concentrations as low as 2% to induce gel formation. Their robust self-assembly capabilities make them particularly suitable for organogels, where they demonstrate enhanced performance in non-polar solvents [38].

Polymeric high molecular weight gelators (HMWGs), with molecular weights exceeding 2 kDa, include polysaccharides and proteins that form gels at concentrations below 2%, relying on physical or chemical interactions [36]. Their ability to form structured networks with water makes them valuable hydrocolloids, with gelation properties influenced by molecular architecture, charge distribution, and interactions with food components. These attributes are critical for modifying food texture, enhancing stability, and enabling controlled release mechanisms [4,10,12,14,18,39,40].

Thus, this review focuses specifically on natural biopolymers and their applications in food systems, where their structural and functional attributes are particularly valuable. Polysaccharides can be classified based on their origins: HMWGs can be categorized into natural biopolymers, such as polysaccharides and proteins, obtained from diverse sources, including plants, algae, and microbes, as well as synthetic polymers like polyvinyl alcohol and carbopol.

Based on information obtained from the bibliometric analysis, we also highlight the following point: other polysaccharides derived from seaweed include agar and carrageenan, which are extracted from red algae such as *Gelidium* and *Gracilaria*. These form strong gels that remain solid at room temperature, making them suitable for jelly candies, dessert gels, and vegetarian gelatine substitutes [41,42,43]. Carrageenan, sourced from red seaweeds like *Chondrus crispus*, exists in multiple forms (kappa, iota, lambda), with distinct gelation properties depending on ion interactions. For instance, kappa-carrageenan forms brittle gels with potassium or calcium ions, whereas iota-carrageenan produces elastic gels with calcium ions. Carrageenan’s are commonly used in dairy products and plant-based meat gels to provide a creamy yet firm texture [44].

In addition, sustainable alternatives such as Seed-based polysaccharides are being promoted. These include guar gum (*Cyamopsis tetragonoloba*), locust bean gum (*Ceratonia siliqua*), flaxseed gum (*Linum usitatissimum*), and chia seed mucilage (*Salvia hispanica* L.), all of which exhibit exceptional water-binding capacity, emulsifying potential, and textural improvement capabilities [39].

Among these materials, chia seed gum (CSG)or mucilage stands out for its promising functionality in bigel systems, combining hydrogels with oleogels structured using glycerol monostearate (GMS). CSG improves network strength, elevating the storage modulus (G’), which enhances gel elasticity and structural integrity. Additionally, it stabilizes hydrogel-oleogel matrices, presenting a viable alternative to animal-derived gelators like gelatin and whey protein concentrate (WPC) [45].

Its biodegradable and sustainable nature aligns with clean-label and health-conscious food trends, offering a natural substitute for synthetic stabilizers and animal-based gelators in hydrocolloid food systems.

#### 2.1.4. New Material Developments: Bigels with Polysaccharide Gums for Improved Functionality

Polysaccharides are composed of monosaccharide units joined by O-glycosidic linkages. Their diverse physical properties (e.g., solubility, flow behaviour, gelling potential, surface, and interfacial properties) and functional properties (e.g., as stabilizers, thickening and gelling agents, crystallization inhibitors, and encapsulating agents) result from their structural diversity. Natural sources of polysaccharide gums include storage materials, cell wall components, exudates and extracellular substances from plants or microorganisms [46].

**Figure 4:**
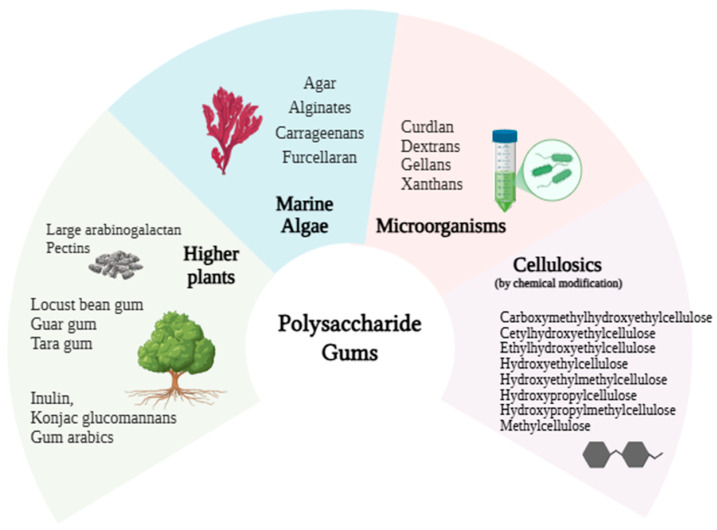
Classification of polysaccharide gums. (Created in BioRender (https://app.biorender.com; accessed on 18 March 2024): Scientific Image and Illustration Software).

Nowadays, polysaccharide gums are utilized in biphasic systems due to their rheological properties, as well as their characteristics of biocompatibility, biodegradability, stability, non-immunogenicity, and non-teratogenicity, which make them suitable for biomedical and food applications. The classification is based on the source, including marine algae, microorganisms, and higher plants (Figure 4). Regarding their structure, they are divided into the following: (i) linear, unbranched molecules (carrageenan’s; alginates); (ii) linear with short branches (guar and xanthan gum); and (iii) branch-on-branch (gum arabic) [47,48]. Hence, these molecules have a strong affinity for water and hydrate readily, showing the ability to gel and/or thicken aqueous systems. However, the systems’ rheological properties depend on the gum type, concentration, process temperature, and component ratios.

Plant-derived polysaccharides, such as starch, pectin, and seed-derived gums, act as natural hydrocolloids with thickening, emulsifying, and gelling properties, widely used in food formulations [18,40,41,42,43,44,45,46,47,48,49,50]. Pectin hydrogels form three-dimensional networks with hydrophilic properties, offering softness, flexibility, and biocompatibility. These features make them ideal for applications in dairy products, fruit-based gels, and encapsulation systems for bioactive compounds [51].

Microbial polysaccharides, such as xanthan gum and gellan gum, are fermentation-derived hydrocolloids with unique functional characteristics. Xanthan gum, produced by Xanthomonas bacteria, functions as a thickener with stable viscosity across varying pH and temperature conditions. When combined with locust bean gum, it forms elastic gels [52]. Gellan gum, synthesized by *Sphingomonas elodea*, forms clear, brittle gels at low concentrations, making it indispensable for applications in plant-based dairy alternatives, confectionery, and structured water-based products [40,50].

These dual systems, with modular and multifunctional properties, enable the encapsulation of both hydrophilic and hydrophobic molecules, leveraging the synergistic effects of gelling agents or gelators and the ratio of both systems to enhance physical, mechanical, and encapsulation stability.

As demonstrated in Table 1, various studies have investigated the oleogel-to-hydrogel ratio, revealing that increasing the oleogel fraction significantly influences the cohesiveness, firmness, consistency, and viscosity index, while also reducing hardness and facilitating a more distinct gel–sol transition [53,54].

Previously listed features also promote the fabrication of bigels that can serve as delivery matrices for both hydrophilic and lipophilic compounds. Examples include sterculia and poly (AAm)-based bigels for gastrointestinal drug delivery and xanthan gum/guar gum matrices for antioxidant encapsulation.

In the context of functional food systems, bigels have been utilized to enhance organoleptic and nutritional attributes by incorporating natural pigments and antioxidants. Lutein, a lipophilic antioxidant, has been efficiently encapsulated within a xanthan gum/guar gum–sunflower oil matrix, retaining significant antioxidant activity under simulated gastrointestinal conditions (ABTS: 26.28 µg GAE/g; FRAP: 89.60 µg GAE/g) [21]. Furthermore, the inclusion of gelling agents such as κ-carrageenan at concentrations above 2 wt.% contributes substantially to the structural reinforcement and viscoelastic stability of the gel matrix [10].

These systems also support advanced manufacturing techniques such as 3D food printing and can mimic conventional dairy creams for low-fat or plant-based alternatives. Structurally, formulations with over 70% hydrogel improve uniformity and mechanical integrity [10].

Other recognized natural polysaccharides for bigels formulation are cellulose and starch. These polysaccharides exhibit structural properties that confer stability, physical integrity, and favourable storage characteristics. Both are inherently insoluble polysaccharides; however, through chemical modification known as derivatization, their water solubility can be altered. Additionally, certain cellulose derivatives, including carboxymethyl cellulose [14], hydroxypropyl methylcellulose [55], and hydroxyethyl cellulose [48], have been utilized in the formulation of hydrogels for bigels synthesis, as observed in Table 1. Overall, bigels offer a versatile and tuneable platform for food engineering, nutraceuticals, and biomedical delivery.

**Table 1:** Different applications of the polysaccharide gums in the bigels.

Application	Bigel Ratio (Hydrogel/Oleogel)	Contributions	Ref.
Drug delivery	Sterculia gum and poly (Aam)/Olive oil and sorbitan monopalmitate): 95:5; 90:10; 85:15; 80:20; 75:25	Bigel formulations (BG2, 90:10) can be proposed for gastrointestinal drug delivery systems due to their hemocompatible, nonhemolytic, mucoadhesive, antioxidant, and viscoelastic nature.	[27]
	Tamarind gum (TG) with a hydroethanolic solution Stearic acid and rice bran oil: 0:30; 6:24; 18:12; 30:0	Diffusion of the hydrophilic drug within the formulation was enhanced significantly in a composition-dependent manner as the TG hydrogel ratio was augmented (6:24, 18:12, and 30:0).	[56]
Fat replacer	Locust bean gum and κ-carrageenan (1:1 ratio) at different concentrations (0.5–2.5 wt.%)/ Sunflower oil and glyceryl monostearate: 50:50; 60:40; 70:30; 80:20; 90:10	Structural matrix elevated in formulations starting at 70 wt% of hydrogel fractions. The HG: OG ratio and biopolymer concentration (above 2 wt.%) influenced the microstructure.	[10]
	Sodium alginate and carboxymethylcellulose Beeswax with canola oil (BW-O): 50:50	BW-CMC presented a slightly lower peroxide value. BW-CMC and BW-ALG showed a higher transition temperature than BW-O.	[14]
	Xanthan gum and Guar gum/Ethylcellulose Sunflower oil: 75:25; 50:50; 25:75	The lutein release during simulated gastrointestinal digestion was 83.2% for bigel (25:75), and the antioxidant activity was ABTS: 26.28 μg GAE/g; FRAP: 89.60 μg GAE/g.	[21]
	Xanthan gum (XG) Spirulina platensis protein nanoparticles (SPNPs) Sunflower wax: 80:20; 60:40; 50:50; 46:54; 44:56; 42:58; 40:40; 20:80	Compared to bigels made of wax-based oleogel, these bigels (O/W, semi-bicontinuous, and W/O types) showed promising printability even with only 5 weight percent SW in OG. For 3D printing, semi-bicontinuous bigels with an OG fraction of 56% was appropriate.	[57]
3D printing	Beeswax: Gellan gum Oleogel: <62%; 62–68% and >70%	A protocol for directing the creation of bigels for 3D meals that have delicate shapes and modified physical characteristics.	[32]
	Sodium alginate into a whey protein nanofiber solution/whey protein isolate -xanthan gum, corn oil emulsion: 80:20; 70:30; 40:60; 50:50	The sensor responses of the 25% BG4 samples were nearly identical to those of the 100% cream. The textural properties of the 50% BG4 variant closely resembled 100% cream in most attributes.	[53]
	Agar (AH) and Gelatin (GH)/OG with 5% beeswax: 95:5; 90:10; 80:20	10 and 20% OGs concentrations in agar allowed the synthesis of bigels with microstructural and viscoelastic properties for printing parts with excellent surface quality, more minor dimensional deviations, and good reproducibility.	[54]
	Hydroxypropyl methylcellulose (HPMC) Beeswax (10%) and (1%) W/O bigels (60% and 80%)	W/O bigels with 60% oleogel content displayed great print integrity in all 3D printing procedures. TPA tests showed that the extrusion of the printing process had a strong destructive effect on W/O and semi-bicontinuous type bigels, but not on O/W bigels.	[55]

#### 2.1.5. Developing Oleogel/Bigel Systems Incorporating Novel Waxes and Oils

The most influential studies on using plant-based waxes and oils in bigel systems are presented in Table 2. These are the top 10 articles out of 157. As there is so much literature, only 10 highly relevant articles are analyzed in Table 2. These articles show current research directions and methodological approaches in the field. There is growing interest in plant-derived components for their abundance, biocompatibility and functional versatility [58]. In this sense, polysaccharides extracted from seeds and vegetable oils are complemented for constructing biphasic networks, and are used to create thickening, emulsifying and gelling properties [18]. They also enhance structural integrity and phase stability in bigels, as demonstrated in systems formulated with chia seed gum (CSG) [45]. In addition, vegetable oils are incorporated into bigel systems to create the lipophilic phase, allowing for the structuring and encapsulation of fat-soluble bioactive compounds. Their inclusion also contributes to desirable properties such as spreadability, crystallinity and thermal stability, making them ideal candidates to replace traditional solid fats in food formulations [4,12,14,39,45].

Plant-based waxes like candelilla, rice bran, and sunflower, function as oleogelators by structuring vegetable oils into semi-solid matrices through crystalline network formation. These waxes complement vegetable oils and play a vital role in stabilizing the oleogel phase of bigels, enhancing their functionality for various food applications [4,6,15,18,32]. An emerging trend in bigel research is their application in 3D food printing, where plant-based waxes enable stable, printable bigels with tailored rheological and structural properties [11,17,32,33,54,57]. This opens new possibilities for the design of nutritionally enhanced, complex food products using sustainable ingredients.

## 3. Conclusions

This study underscores the versatility of polysaccharide-based bigels as sustainable biphasic systems with broad applications across food, pharmaceutical, and biomedical fields. By integrating hydrogels and oleogels, bigels facilitate the encapsulation of both hydrophilic and lipophilic compounds, enhancing structural stability and bioactive delivery.

In this context, bibliometric and principal component analyses identified the four key research domains: structural applications in food and biomaterials; bioactive encapsulation and release; therapeutic delivery systems; and rheological and stability assessments. These findings highlight the interdisciplinary and rapidly evolving nature of bigel research.

Additionally, emerging materials such as seed-derived polysaccharides, chia gum, for instance, have been shown to improve bigel performance by enhancing elasticity, biocompatibility, and gelling capacity, while natural waxes and vegetable oils reinforce the oleogel phase. Collectively, these components contribute to the development of functional, clean-label formulations that align with innovations such as 3D food printing.

Ultimately, bigels present a promising platform for future advancements, with bibliometric analysis serving as a valuable tool for guiding research priorities and formulation strategies.

## 4. Materials and Methods

The information search was conducted using a predefined algorithm with keywords and multiple Boolean combinations (AND, OR). These combinations were applied to the titles, keywords and abstracts of the extracted documents, resulting in the following query: TITLE-ABS-KEY (bigel) AND (polysaccharides OR seed OR hydrocolloids OR (vegetable AND oil) OR waxes) AND PUBYEAR 2015 AND PUBYEAR 2026 AND (LIMIT-TO (DOCTYPE, ar)).

The search was performed and last updated 5 March 2025, using the Scopus database. The extracted information found was exported to a CSV file for analysis. To ensure thematic relevance, the titles, abstracts, and keywords of all documents were meticulously reviewed, covering a decade from 2015 to 2026.

To identify the relevance of the main subfields, Principal Component Analysis (PCA) was employed as a statistical technique for synthesizing information or reducing dimensionality (number of variables) within datasets containing many variables. This approach aimed to reduce the dataset to a smaller number of variables while minimizing information loss.

A quantitative analysis was carried out using author keywords, standardized through the merging synonyms, eliminating irrelevant terms and curating a controlled vocabulary to ensure consistency. A binary presence matrix was then constructed: each row represented an article, and each column corresponded to a specific keyword, marked with a value of 1 if present and 0 if absent. To increase statistical robustness, keywords with very low frequency (i.e., appearing in fewer than two articles) were excluded.

PCA was performed using Minitab V.18.1 (Minitab, Inc., State College, PA, USA) to identify dominant research themes and keyword relationships. Given the binary nature of the dataset, the correlation matrix was used instead of the covariance matrix to avoid singularity problems. This approach enables effective dimensionality reduction while preserving significant patterns of variance.

A biplot was generated to visualize keyword associations, alongside a loading plot to determine the most influential terms contributing to each principal component. Additionally, a bibliometric map of the keywords was created using co-occurrence analysis. Only terms appearing in at least five documents were included, based on a total dataset of 1540 keywords. The mapping process was conducted using VOSviewer software V. 1.6.15 (Leiden University, Leiden, The Netherlands), a bibliometric network visualization tool developed by the Centre for Science and Technology Studies (CWTS).

## Figures and Tables

**Table 2:** Trend Analysis of Bigels Containing Different Waxes.

Bigel (Hydrogel/Oleogel)	Applications	Aim	Key Findings (Contributions)	TC/Journal IF	Country/Year	Reference
Sodium Alginate and Carboxymethylcellulose/Beeswax and canola oil	Replace saturated and trans fats in cookies	Evaluate the role of hydrogel type in the development of bigels to be used as SFAs and TFAs replacers in cookies.	Bigels showed lower peroxide values than pure oleogels and canola oil, indicating improved oxidative stability and suitability as saturated fat replacers in bakery applications.	59/5	Chile/2022	[14]
Agar/Beeswax and glyceride monooleate and sunflower oil	Sensor for intelligent food packaging	Develop a colorimetric sensor for volatile amines using anthocyanins encapsulated hydrogel-in-oleogel bigel for monitoring beef and salmon freshness.	The bigel protected anthocyanins and enabled a 3D-printed freshness sensor that changed colour (red to purple) in response to trimethylamine, indicating meat/fish spoilage.	61/11	China, UK/2022	[33]
κ-carrageenan-xanthan gum/Beeswax and corn oil	3D food printing	Investigate the 3D printability, rheological properties, and microstructure of bigel inks containing different concentrations of beeswax oleogel.	Increasing oleogel content improved printability, viscosity, and mechanical strength. BG5 (80% oleogel) showed the best performance for 3D food printing with strong self-support and fast recovery.	110/11	China/2022	[34]
(High acyl gellan gum/Beeswax and Soybean Oil)	Develop bigels as a semi-solid vehicle for lycopene delivery	Fabricate novel bigels as a semi-solid vehicle for lycopene delivery	Effective bigel system for lycopene delivery; release and structure depended on oleogel content. Functional fat replacer.	139/8.5	China/2021	[40]
Hydroxypropyl mehhylcellulose (HPMC)/Beeswax and Soybean oil	3D food printing	Synthesize and characterize bigel systems with semi-solid properties, combining beeswax oleogels and HPMC hydrogels in varying ratios using PGPR as an emulsifier.	The W/O system (60% oleogel) showed superior mechanical and 3D printing properties. Phase inversion was observed as oleogel content increased. The system showed potential as a solid fat replacer and for customized food design.	65/11	China/2023	[55]
Xhanthan gum/Sunflower wax and Soybean oil	3D food printing	Examine bigel system formation through phase inversion process, varying oleogel/hydrogel ratios to enhance 3D printability and facilitate personalized food design	Spirulina platensis protein nanoparticles improved emulsion stability, phase control, and printability, enabling their use in clean-label fat replacer.	48/11	China/2023	[57]
κ-carrageenan/Beeswax or Glycerol Monosterate and Corn oil	Novel functional products	Develop novel bi-phasic gel systems incorporating a hydrogel and an oleogel, and investigate the influence of different oleogelators on the structures of the bigels.	Higher oleogelator content increased mechanical resistance, supporting their use for bioactive delivery in food.	48/7	China/2022	[59]
Polymeric hydrogel (not specified)/Candelilla wax vs. 12-gydroxstearic acid	Topical delivery system for vitamin E (cosmetic/pharmaceutical)	Understand the structure, rheology and stability of bigels and their corresponding emulsions in the presence of vitamin E as a model of lipophilic drug.	The oleogelator type affected the texture and thixotropy. Vitamin E had little impact on bigel stability.	47/5.4	Brazil/2021	[60]
Fish gelatin/Candelilla wax and high oleic sunflower oil	3D printed food systems for bioactive delivery (quercetin, catechin)	Produce 3D-printed bigels based on candelilla wax oleogel and gelatin hydrogel as a delivery system for hydrophilic and lipophilic bioactives.	Developing bigels as 3D printed food with potential in co-delivery of hydrophilic and lipophilic bioactives by adjusting oleogel/hydrogel ratios and emulsifiers used.	46/11	China, USA/2023	[61]
Fish gelatin/Candelilla wax and high oleic sunflower oil	3D food printing	Explore bigel applications in 3D printing by formulating food bigels using candelilla wax-based oleogel and gelatin hydrogel at varying ratios with different emulsifiers and analyzing their effects on physical properties and printability.	Bigels with monoglyceride at a 7:3 oleogel/hydrogel ratio showed the best mechanical strength and 3D printability, while PGPR caused phase separation and weak gels.	42/11	China, USA/2023	[62]

## Data Availability

Data presented in this study are available upon request from the corresponding author.
